# Bandage after Labor

**Published:** 1891-05

**Authors:** 


BANDAGE AFTER LABOR.—Before the Obstetrical Society of London, Dr. Herman considered the use of the binder or bandage during the lying-in period, and concluded that its sole utility consisted in the comfort it gives the patient. He did not think it had any effect in keeping the waist measurement small, and so preserving the figure of the patient. To counteract the injurious effects of the sudden lowering of the intra-abdominal pressure it should be applied at the moment that evacuation of the uterus takes place. Dr. Gervis said that patients wanted it not so much because it might influence the size of the waist, but for the support it gave to the lower abdomen and its effects in checking any tendency to undue fullness there afterwards. It promotes uterine action and checks haemorrhage. Non-use of the binder leads occasionally to "pendulous belly," and its consequences. When properly adjusted it promotes involution.—*Med. Herald.*

